# Integrating use of point-of-care circulating cathodic antigen rapid diagnostic tests by community health workers during mass drug administration campaigns to improve uptake of praziquantel treatment among the adult population at Kome Island, North-Western Tanzania: a cluster randomized community trial

**DOI:** 10.1186/s12889-018-5732-y

**Published:** 2018-07-06

**Authors:** Humphrey D. Mazigo, John H. Amuasi, Isaac Osei, Safari M. Kinung’hi

**Affiliations:** 1 Department of Medical Parasitology, School of Medicine, Catholic University of Health and Allied Sciences, P.O. Box 1464, Mwanza, Tanzania; 2 African Research Network for Neglected Tropical Diseases, Kumasi Center for Collaborative Research in Tropical Medicine, KNUST, PMB UPO, Kumasi, Ghana; 3 National Institute for Medical Research, P.O. Box 1464, Mwanza, Tanzania

**Keywords:** Intestinal schistosomiasis, *Schistosoma mansoni*, Point-of-care circulating cathodic antigen tests, Praziquantel, Community health workers, Tanzania

## Abstract

**Background:**

The major drawback of the community-based mass drug administration (MDA) approach against schistosomiasis is that treatment is offered blindly without testing for the targeted infection. This partly contributes to the low treatment coverage. One approach to overcome this limitation is to introduce a diagnostic component in the treatment approach. This will improve drug uptake and compliance to treatment. This study is conducted to assess the feasibility and acceptability of integrating point-of-care Circulating Cathodic Antigen (POC-CCA) test to community-based directed MDA in improving treatment coverage and compliance with treatment among adults.

**Methods:**

This is a randomized control community trial in which 30 clusters were randomly assigned to either an intervention or control arm to evaluate two interventions on treatment coverage and compliance with treatment. In each cluster, 150 adult participants were enrolled. Community Health Workers (CHW) in both arms were trained on all aspects of praziquantel (PZQ) distribution and management of mild side effects. In the intervention arm, CHWs had additional training on how to use POC-CCA to diagnose intestinal schistosomiasis. In the intervention arm, participants were tested using POC-CCA test for presence of intestinal schistosomiasis and treated based on test results, while in the control arm, participants were treated with PZQ without testing. The primary outcome measure was the proportion of participants provided with PZQ between the two arms and geographical clusters. Secondary outcomes were prevalence of *S. mansoni* infection based on the POC-CCA test conducted by CHWs, ability of CHWs to use the POC-CCA test accurately and safely and community acceptability of the POC-CCA test results from CHWs. Both quantitative and qualitative techniques have been used to collect data at study endpoint.

**Discussion:**

The study will generate evidence on the importance of integrating a diagnostic component into the community directed MDA conducted by CHWs. Findings will generate discussion on the current MDA policy and practice in Tanzania.

**Trial registration:**

PACTR201804003343404 (25/4/2018).

## Background

Schistosomiasis infection is a neglected tropical disease which is highly prevalent in Sub-Saharan Africa (SSA), and is a major cause of public health burden and economic impact [1, 2]. Within the SSA region, two major forms of schistosomiasis-causing species exist: *Schistosoma mansoni* and *Schistosoma haematobium* which cause intestinal and urogenital schistosomiasis respectively [1]. Of the estimated 258 million people infected with schistosomiasis, 90% (192 million cases) occurs in SSA and 779 million people reside in areas with a high risk of schistosomiasis transmission [2, 3]. In Tanzania, because of the wide distribution of schistosomiasis, the entire population of approximately 45 million people remain at risk of the disease, and in 2012 it was estimated that 51.5% of the population was infected [2, 4]. In Tanzania, *S. mansoni* has remained a major public health concern, especially among communities living along and on Lake Victoria islands, in the north-western region [5, 6]. Disabling morbidities associated with schistosomiasis infection include anaemia, malnutrition, impaired development in childhood due to prolonged inflammation and hepatosplenic morbidities characterized by hepatomegaly, splenomegaly and periportal fibrosis in adulthood [7, 8].

The World Health Organization’s (WHO) strategic plan of 2012 proposed scaling up of mass drug administration (MDA) as a means of controlling schistosomiasis by 2020 [9]. Current control of schistosomiasis in Tanzania is based on preventive chemotherapy using praziquantel, the drug of choice, through mass drug administration programs targeting school-aged children in their school environment [10]. Targeting anthelminthics at school-aged children capitalizes on the fact that the heaviest burden of infection is found in this segment of population [10]. However, the major drawback of the school-based approach is that it does not include non-school-going children and other members of the community [11, 12]. In addition, anti-schistosomiasis drugs are not always available in many of the health facilities in endemic areas [13]. From the public health perspective, any infected individual, regardless of age, may represent as a source of infection to the rest of the population, including treated school children [10]. Thus, to achieve a significant reduction in disease burden, every member of the schistosomiasis endemic areas must be reached by the control interventions [11, 12]. The WHO, having knowledge of this, initiated community directed treatment programs [10], in which individuals selected by communities are trained and participate in distributing drugs to other members of the community not covered by the school based program [10].

To increase treatment coverage, community directed treatment has been implemented as an alternative approach for controlling schistosomiasis in a number of African countries to improve access to treatment for community members not included in mass drug administration program [14, 15]. Through this approach, the WHO recommends 75% treatment coverage at community level [9]. In this approach, Community health workers (CHWs) are actively involved in delivering treatment [14, 15]. However, in many of the community directed interventions against schistosomiasis, treatment coverage does not reach the WHO recommendation of 75% [9]. In Uganda for example, the uptake was 50% [16]. Multiple factors have been described to affect the performance of community directed treatment campaigns in schistosomiasis endemic areas [17–19]. Among the major drawback of this approach, is that treatment is offered blindly without any diagnosis of the targeted infection. This may affect community compliance and uptake of treatment, especially among adults. Alternatively, participants who are diagnosed of the targeted infection are more likely to accept and comply with the treatment compared to those treated without diagnosis. In malaria, use of rapid diagnostic testing by community health workers to diagnose malaria increased adherence to treatment [20, 21]. With the more recent easy availability of praziquantel, coupled with widespread escalation of schistosomiasis control using MDA in sub-Saharan Africa, a decline in schistosomiasis infection prevalence is expected. This expected decline could be accelerated by selective mass treatment rather than mass treatment without establishing the status of the infection using any diagnostic technique particularly among adults living in endemic areas [22, 23]. The integration of a diagnostic component into the community-based MDA using trained community health workers is also likely to reduce overall costs.

Point of care Circulating Cathodic Antigen (POC-CCA) test is a simple rapid test, easy to perform and interpret, as the results based on colour change [24]. The test has been recommended by the WHO for screening of intestinal schistosomiasis in endemic areas [24]. The simple nature of the POC-CCA test in terms of testing and interpreting the results based on color change, offers an opportunity to train even unskilled persons with minimal levels of education to perform the test and give treatment based on the test results. However, despite the availability of this opportunity to train community health workers to diagnose and treat schistosomiasis using the POC-CCA tests, no single study has assessed the use of these tests at community level using community health workers. We therefore have the main question: “does integration of rapid diagnostic test for intestinal schistosomiasis diagnosis performed by community health workers increase the uptake and compliance with treatment in adult populations”?

The pre-existing community health workers participating in MDA campaign at Kome Island offers an opportunity to improve the community-based MDA campaign by introducing a diagnostic component into the program to increase confidence of participants in the campaign and increase uptake of treatment [14]. In addition, the wide use of community health workers to diagnose intestinal schistosomiasis could facilitate diagnosis of the disease in settings with limited health personnel, health facilities and increase treatment coverage. Such an approach would help reduce the burden and transmission of intestinal schistosomiasis in the targeted communities. In this context, the main objective of the present study was to assess if introducing point-of-care Circulating Cathodic Antigen (POC-CCA) rapid test to community based mass drug administration through community health workers will increase access, compliance and coverage to treatment among adult individuals at Kome Island in North-Western Tanzania, an area on the Lake Victoria highly endemic for schistosomiasis.

### Hypothesis, aims and objectives

In this study, we hypothesize that integrating point-of-care Circulating Cathodic Antigen tests in community directed mass drug administration campaigns increases the uptake, compliance and coverage of schistosomiasis treatment among adult individuals above the 75% recommended by the WHO [9]. The general objective of this study was to assess whether integrating point-of-care Circulating Cathodic Antigen rapid diagnostic testing by community health workers during a mass drug administration campaign increases uptake of praziquantel treatment among adult population. To address this objective, this study was conducted at Kome Island located in Buchosa district, northwestern Tanzania, an area around the Lake Victoria highly endemic for schistosomiasis. Clusters (service areas served by community health workers) were randomised to receive praziquantel drug without diagnosis or with diagnosis of intestinal schistosomiasis using POC-CCA test and to receive treatment based on the test result.

Primary objective:-

1. To determine the impact of using point-of-care the circulating cathodic antigens rapid diagnostic test by community health workers to diagnosed intestinal schistosomiasis on the uptake of praziquantel treatment among adult individuals diagnosed with *Schistosoma mansoni* infection.

Secondary objectives:-

1. To assess community health worker’s ability to use point-of-care the Circulating Cathodic Antigen rapid test safely and accurately in diagnosing *Schistosoma mansoni* infection in adult individuals and associated factors.
2. To assess community acceptability and attitude towards the use of a point-of-care diagnostic tool for *Schistosoma mansoni* infection within the context of community directed intervention among adults.
3. To document the experience of community health workers after participating in diagnosis of *Schistosoma mansoni* infection using the point-of-care Circulating Cathodic Antigen rapid test.

## Methods

### Study design and area

**Fig. 1:**
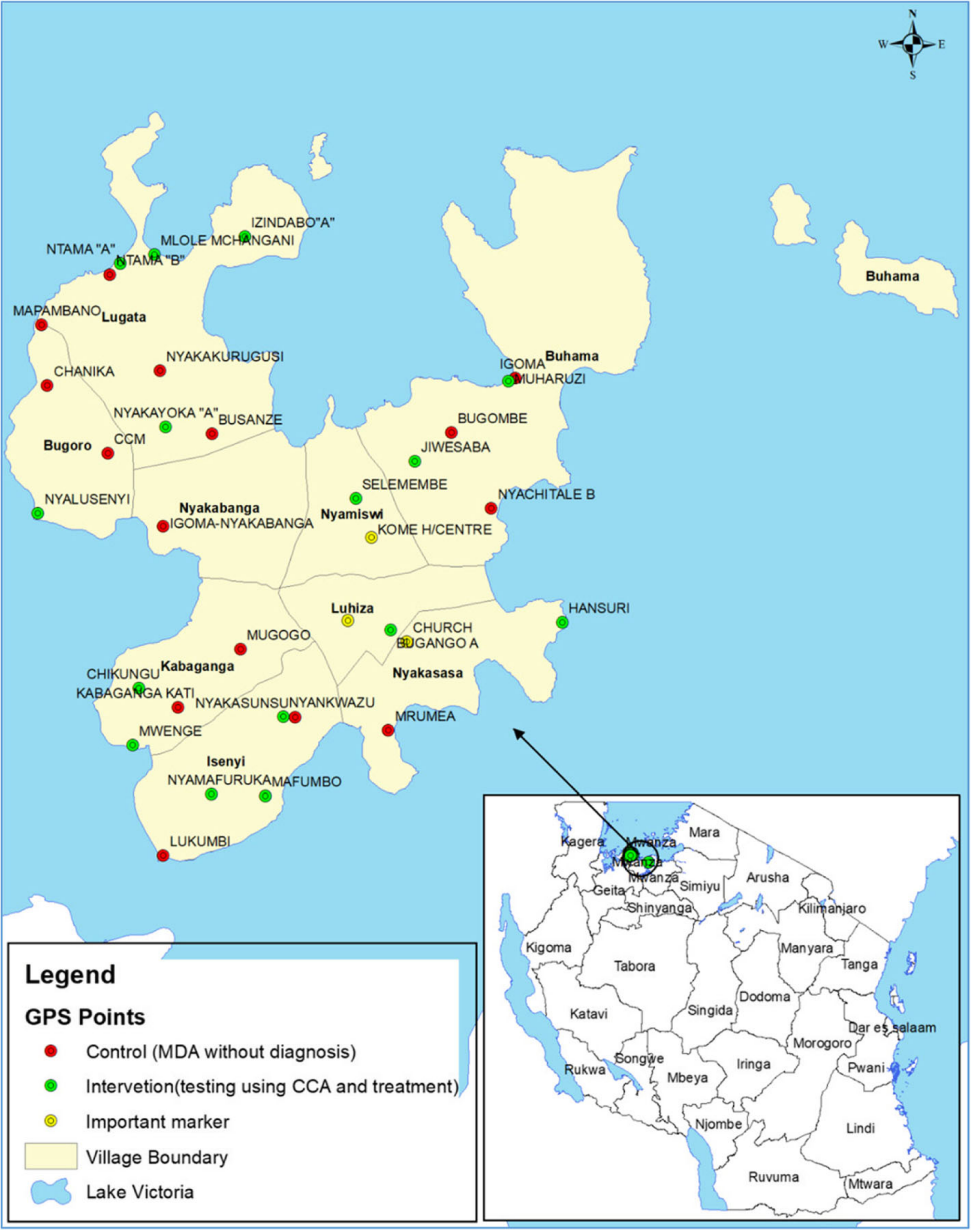
Showing allocation of clusters (sub-villages) included in the study arms at Kome Island, North-western Tanzania. (Map was created using ExpertGPS (version 10.3, ESRI, Inc., Redlands, CA) using layers from ESRI data and Maps 10.1)

A cluster randomized two-arm community trial was conducted, in which clusters were defined as the service population of the community health worker and it involved 30 clusters. The study was conducted at Kome Island located in Buchosa District, North-Western Tanzania (Fig. 1). The Kome Island was purposively selected due to high prevalence of *S. mansoni* infection and existence of a strong CHWs system involved in previous community-based schistosomiasis control projects. According to the population census of 2012, the Kome Island has a population of 38,062. Administratively, it has four wards and 16 villages. It is served by one health centre and two dispensaries. Each village has at least one primary school, with the exception of Isenyi village, which has three primary schools, and four villages with no primary school. The main sources of water are Lake Victoria, natural wells and streams. Schistosomiasis is highly endemic in the area and *Schistosoma mansoni*-related morbidities are quite high among the adult population. Recent records indicate that 42.2% of the adult population had periportal fibrosis, 68% had left liver lobe hepatomegaly and 55.2% had splenomegaly [25].

Schistosomiasis control activities at Kome Island started in 1989 and focused mainly on research and treatment of infected individuals [14]. From 2005 to 2006, a National Schistosomiasis and soil-transmitted helminths (STH) program, with the support of Schistosomiasis control initiatives, implemented a MDA program targeting primary school children for two years [26]. Between 2009 and 2013, integrated community- and school-based treatment programs were implemented in the area [14]. The most recent round of MDA under national Neglected Tropical Diseases (NTDs) control program was carried out among primary school children in late 2015.

### Community health workers

Community Health Workers (CHWs) are an integral part of the health care system in Tanzania [21, 27]. Community Health Workers are appointed by the community among the village members based on minimum criteria that include ability to read and write clearly, to enable record keeping of health interventions which are undertaken in these communities. Depending on the objective of the program, CHWs receive training on health promotion.

At Kome Island, based on previous projects, each of the then 53 sub-villages had two CHWs (a male and a female). However, currently there are 80 sub-villages, and the present study used existing CHWs previously trained on the community-based MDA campaign [14]. There were 54 newly recruited CHWs for 27 sub-villages who took part in this study.

### Trial summary

**Fig. 2:**
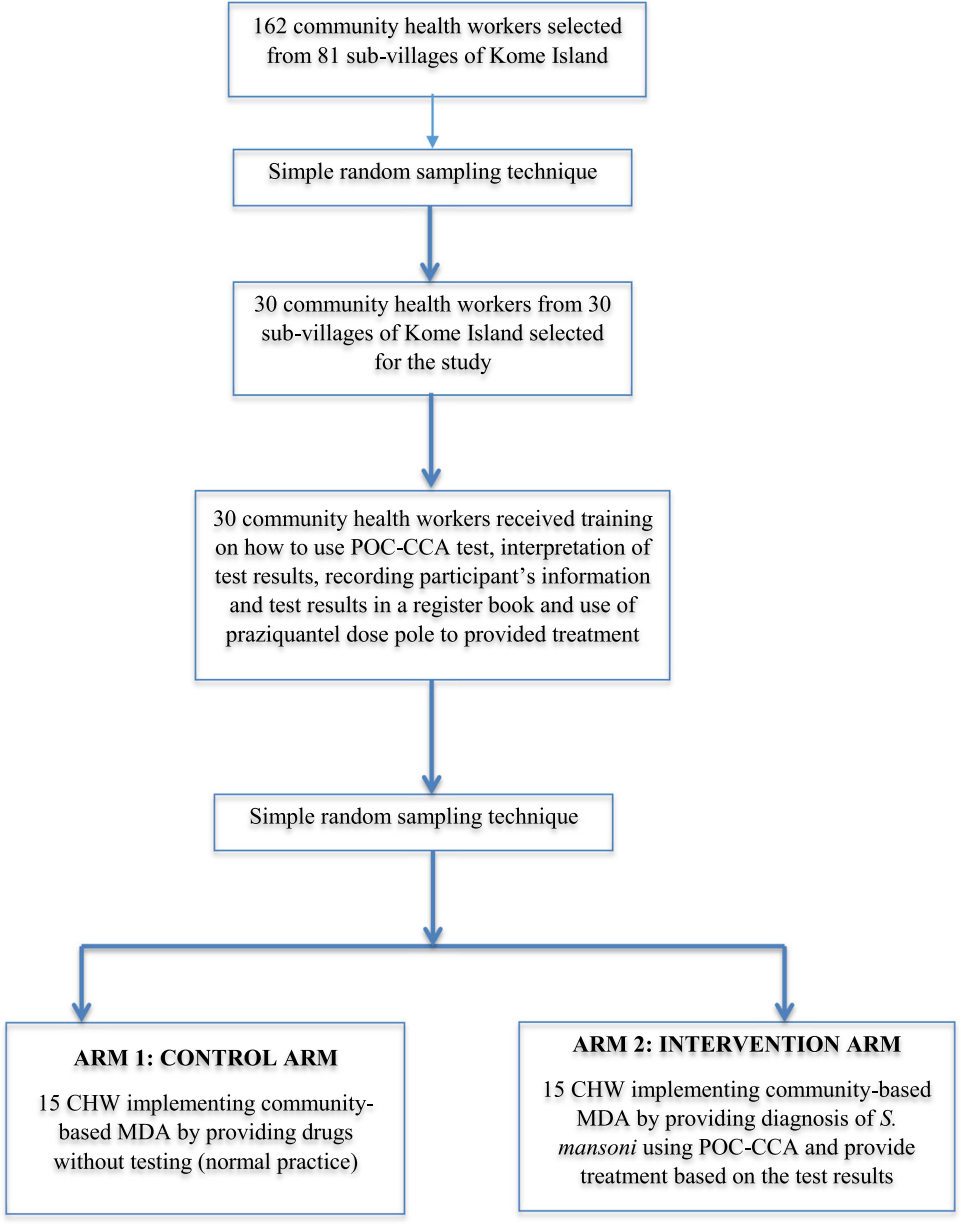
Flow diagram of trial design and number of sub-villages

The study was designed as a cluster randomized two-arm community trial involving randomly selected sub-villages (clusters) at Kome Island. In the intervention arm, community members were tested for intestinal schistosomiasis by CHWs using the POC-CCA test and treated based on the test result (Fig. 2). In the control arm, per current practice, community members received praziquantel treatment from CHWs without being tested. Table 1 shows a summary of the trial.

**Table 1:** Summary of the study profile

Study design	Cluster randomized controlled community trial
Intervention	Community health workers testing for *Schistosoma mansoni* infection using Point-of-care Circulating Cathodic Antigen test before offering treatment
Control	Mass drug administration using praziquantel without testing for *Schistosoma mansoni* infection as per current practice
Primary outcome	Proportion of study participants provided with praziquantel drug (treatment coverage) and geographical cluster (a service area of the community health workers) in the intervention and control arms
Cluster eligibility criteria	Having more than 60 adult individuals served by two community health workers
Inclusion	Individuals aged ≥18 years residing in a study cluster (s), willing to participate and give written informed consent
Exclusion	Pregnant women at the time of treatment, history of adverse reaction to praziquantel and acutely ill at the time of treatment
Sampling eligibility criteria	Residents of the selected clusters or residents of Kome island
Sampling exclusion criteria	Non-resident of the cluster or Kome island as identified by community health workers participating in the study
Sample size per arm	Total sample size (*N* = 4500), Intervention arm (n = 2250) and control (*n* = 2250)

To assess CHWs ability to use the POC-CCA test safely and accurately in the intervention arm, a trained observer (a laboratory technician) visited the study cluster at two, four and six weeks post-training and used a checklist to assess CHWs compliance to safety procedures (Table 2).

**Table 2:** Shows the steps required to correctly and safely prepare a Circulating cathodic Antigen rapid diagnostic test

S/no	The steps required to correctly and safely prepare a circulating cathodic antigen test (Critical steps in noted boldface type)	Score
1	Prepare the diagnostic table (cover it with a plastic cover), assemble new test, pippete and gloves	
**2**	**Put on a pair of gloves**	
3	Provide participants with new urine container (s) and instruct them on how to collect urine sample(s) using the provided container	
4	Recording participants demographic information (name, age, sex and village of residence) in the registry when submitting urine sample (s) at the diagnostic table	
**5**	**Receive urine sample from participant (s) label the container with participants identification number and place it in a sample collection dish**	
**6**	**Take Point-of-Care circulating cathodic antigen test kit (s), label it with participants (s) identification number which is similar to the one written on the urine container**	
7	Place the labelled point-of-care circulating cathodic antigen test kit on the table (level surface)	
**8**	**Take the urine container with identification number similar to the one labelled on the point-of-care circulating cathodic antigen test kit and collect urine sample using the enclosed pipette and pour 1–3 drops of urine sample in a test kit well**	
**9**	**Dispose the used pippete in a waste bucket immediately after pouring the urine sample in a test kit**	
**10**	**Dispense 2–3 drops of clearing buffer if provided in a sample well**	
**11**	**Wait for 15–20 min before reading the POC-CCA test result (either positive or negative)**	
**12**	**Read the result correctly (either negative or positive)**	
13	Record the result of the POC-CCA test in the participant register book	
14	Give the test result (s) to participants, and based on the test result, advise them (either to take treatment if results are positive or no treatment if negative)	
15	Dispose of used gloves, desiccant, wrappers and used test kits.	

The bolded sentences shows the most critical steps during preparation and use of the point-of-care circulating cathodic antigen test

At baseline, a quantitative questionnaire was used to collect data on CHW’s and community member’s (heads of households) knowledge about schistosomiasis, the previous community-based MDA strategy, acceptability to be diagnosed for intestinal schistosomiasis by CHWs and treatment with PZQ without being diagnosed for the targeted infection. At the end of the study, qualitative Focus Group Discussions (FGDs) were conducted to assess the experiences of CHWs from both arms after participating in the trial. FGDs were held with selected community members who participated in the study to assess community acceptability and attitudes towards use of POC-CCA test by CHWs.

Randomization of clusters to either intervention or control arms was based on the population size of the adult individuals served by a community health worker. All CHWs serving clusters with less than 60 adult populations were excluded from the study.

Randomization of the CHWs to receive the POC-CCA test plus PZQ or PZQ without POC-CCA testing was performed using the *ralloc* programme in Stata version 13. Community Health Workers was the unit of randomization and the unit of analysis (because CHW were representing each of the sub-villages they served). Thirty clusters (i.e. service population served by CHW) with ≥60 adult population were selected from all eligible clusters to participate in the study. The selected cluster were at least 7–15 km away from each other, regardless of whether they were randomized to be in the control or intervention arm. The cluster encompassed a radius around the CHW of at least 5 km and allowed a buffer zone of at least 5 km between the boundaries of different clusters. Buffer zones between the sites had no activities undertaken to prevent contamination between clusters.

### Primary outcomes

The primary outcome is the proportion of study participants provided with praziquantel drug (treatment coverage) and geographical cluster (a service area of the community health workers) in the intervention and control arms.

### Secondary outcomes

Secondary outcomes are:-

1. Community Health Workers (CHWs) ability to use the POC-CCA rapid test accurately and safely.
2. Community members acceptability (these are people’s perceptions and attitude on the two-treatment approach) of the POC-CCA test results offered by community health workers.
3. Experience of Community Health Workers on the use of the POC-CCA rapid diagnostic test.
4. Prevalence of *S. mansoni* infection based on the POC-CCA test conducted by Community Health Workers.

### Study population and inclusion criteria

Individuals aged 18 years and older residing at Kome Island were enrolled after written informed consent was obtained. Participants were enrolled in the study if: (i) aged 18 years and above (ii) willing to participate and give written informed consent and (iii) living in the study area. Participants were excluded from the study if: (i) pregnant at the time of treatment (ii) history of adverse reaction to praziquantel and (iii) present with serious conditions at the time of treatment and not deemed fit to take treatment.

### Sample size

The sample size was estimated using a simplified formula by Hayes and Bennet for cluster randomized trials [28], with a power of 80% to detect an absolute difference in proportion of adult individuals participating in mass drug administration of 25% (50% versus 75%) between the two arms with alpha of 5% and assumed coefficient of variation of 0.3616. The coefficient of variation of 0.3616 was used to account for unequal cluster size. In the intervention arm, we take 75% as level of treatment coverage recommended by WHO at community level [9]. A minimum estimated sample of 150 individuals for each cluster for 30 clusters giving an overall total of 4500 individuals (2250 interventions, 2250 controls) were needed to answer the research questions assuming the standard deviation between clusters of 0.226. For this study, cluster was defined as the service population of the community health worker.

### Training of community health workers on how to perform point-of-care circulating cathodic antigen test and use of praziquantel dose pole

**Fig. 3:**
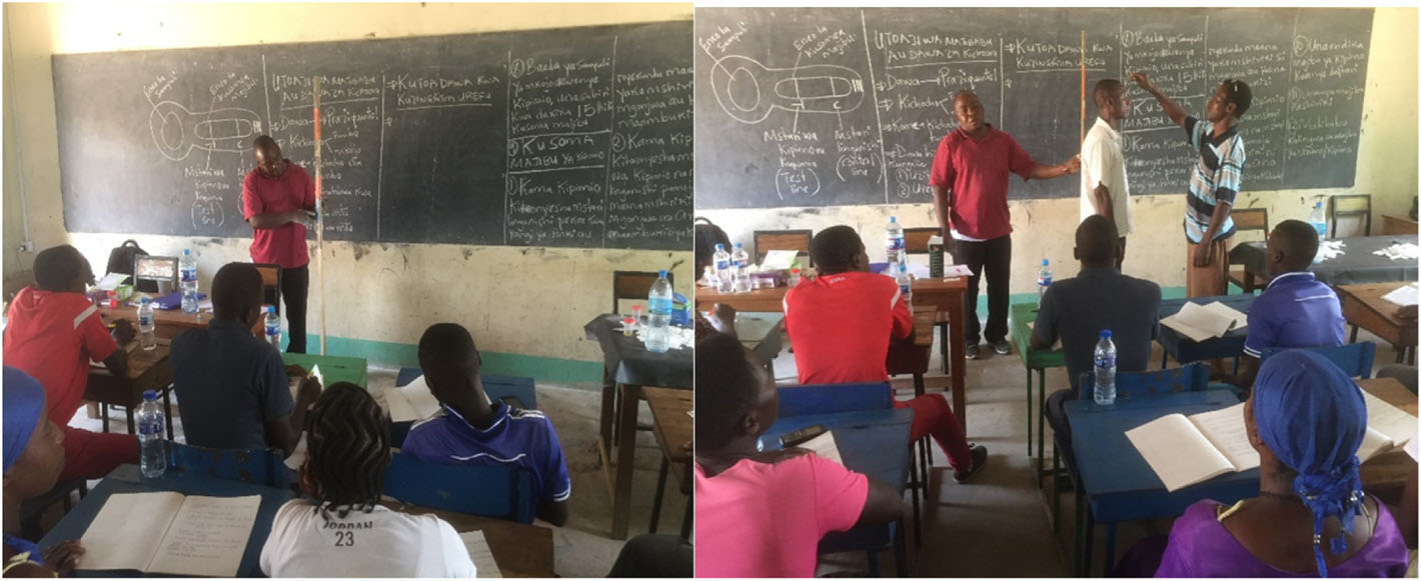
Training and showing community health workers on how to measure height of participant and decide on the number of tablets to give based on height using the Praziquantel Dose pole at Kome Island, north-western Tanzania (The PI teaching CHWs)

Community health workers attended a three-day training on schistosomiasis, covering topics that included epidemiology, the national neglected tropical diseases control program and mass drug administration approach (school-based versus community-based treatment approach), causes, signs and symptoms of intestinal schistosomiasis and standard operating procedures of the study. In addition, CHWs were trained on how to dispense praziquantel drugs using the praziquantel dose pole, based on the height of the study participants and how to record the number of tablets/pills provided to each participant (Fig. 3). The CHWs were also trained on how to handle any side effects observed later or immediately after taking the treatment.

**Fig. 4:**
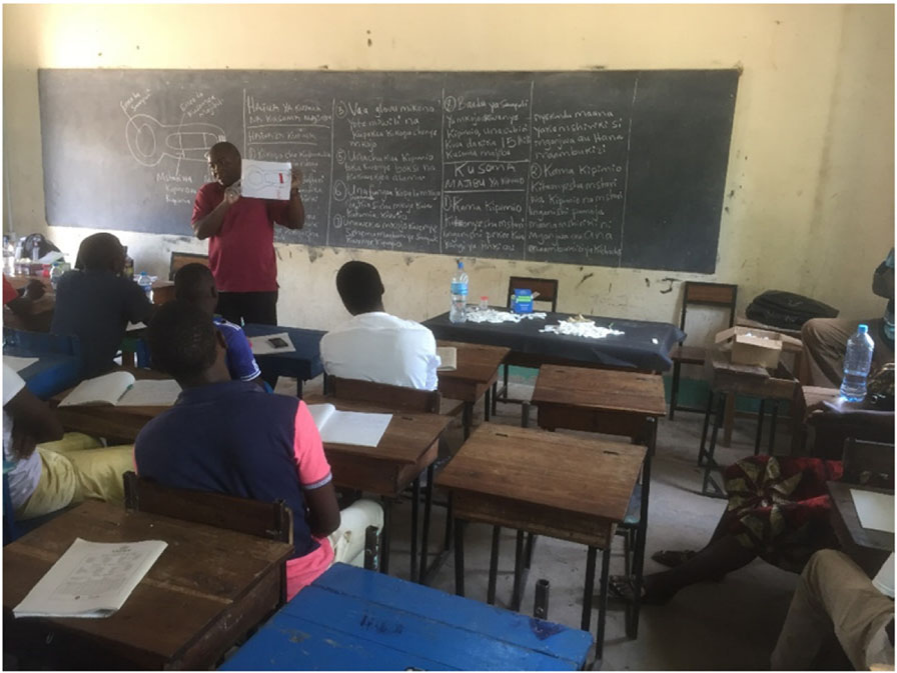
Training community health workers on the principal of Point-of-Care Circulating Cathodic antigen tests at Kome Island, north-western Tanzania (the PI teaching CHWs)

**Fig. 5:**
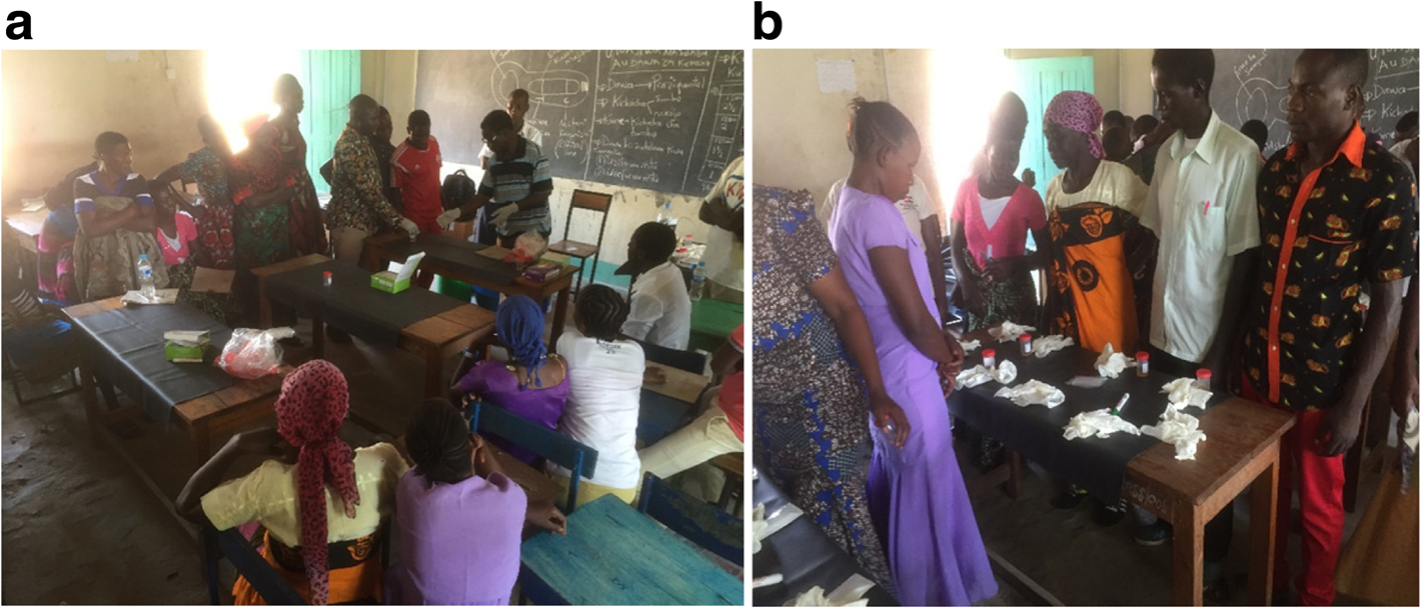
**a** Laboratory technician demonstrating to community health workers on how to follow procedures of performing a POC-CCA test safely and accurately and (**b**) Practical class of community health workers after demonstration class at Kome Island, north-western Tanzania

Community health workers in the intervention arm attended one day of additional training covering how to perform and interpret the rapid test for intestinal schistosomiasis (Fig. 4). The topics covered included; how to request urine samples from participants, adherence to safety measures (wearing gloves before collecting urine containers with urine and washing used urine containers using provided disinfectants), urine safety and handling urine samples after analysis, labelling of the urine containers and test kits with specific study identification numbers, how to add urine sample to the test kit, interpret the result of the test (based on colour changes after 20 mins) and recording the results in the registry booklets provided. The training involved lectures, group discussions and demonstration by a qualified laboratory technician followed by practical sessions (Fig. 5). Community health workers practiced all the areas on which they were trained. After training, CHWs were given a spot examination to assess their ability to interpret the POC-CCA test. Used POC-CCA test kits from a previous study with positive, negative and unused test were given to CHWs for interpretation. The results of the spot examination are shown in Table 3.

**Table 3:** Shows the performance of the spot test of community health workers after training on the preparation and interpretation of the Point-of-Care Circulating Cathodic Antigen tests

Test kit ID	Expert laboratory technician		Community health workers (CHWs)		Total marks for each test kit for CHWs (%)
	Positive	Negative	Positive	Negative	
122	Positive		Positive		100
14		Negative		Negative	100
60	Positive		Positive		100
d16	Positive	Negative	Positive		100
20	Positive			Negative	97
53	Positive		Positive		93
17	Positive		Positive		100
118		Negative		Negative	80
93		Negative	Positive		100
100	New test (unused kit)		New test(unused kit)		70

After the training, all community health workers were provided with praziquantel dose poles, registry booklets, writing materials and praziquantel drugs. In addition, community health workers in the intervention arm were provided with gloves, buckets, urine containers and point-of-care circulating cathodic antigens tests.

### Training of laboratory technicians on how to assess ability of community health workers to use

Two laboratory technicians were trained on how to assess the ability of trained CHWs in the intervention arm to adhere to the safety procedures and standard operating procedures of performing the POC-CCA test. The laboratory technician acted as an observer, visited the study sites (clusters) at two, four and six weeks post-training to assess CHWs using a standardized checklist (steps of test preparation and safety measures) as shown in Table 2. On site re-training was organized for poorly assessed CHWs.

### Data collection and follow-up

#### Recording of demographic information of participants and community mobilization

Trained CHWs in both arms recorded all demographic information of the study participants in the provided booklets. Collected demographic information included age, sex, sub-village of residence and history of taking anti-schistosomiasis treatment.

#### Diagnosis of *Schistosoma mansoni* infection using circulating-cathodic antigen test by community health workers

**Fig. 6:**
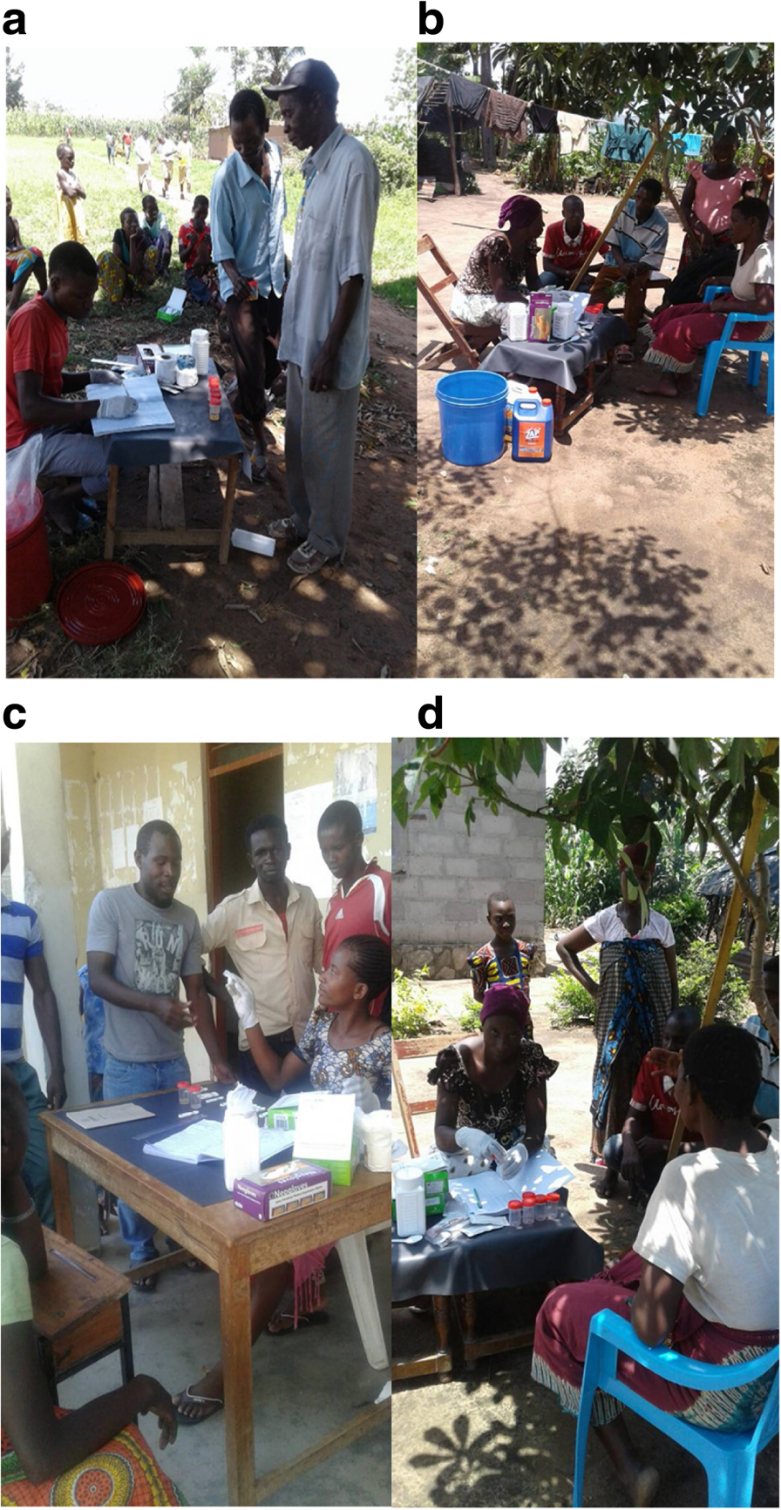
Community health workers in the intervention arms (**a**, **b**, **c**, **d**) were involved in diagnosis of intestinal schistosomiasis using the POC-CCA test in different study clusters at Kome Island, north-western Tanzania

Trained CHWs in the intervention arm diagnosed study participants for *S. mansoni* infection using a commercially available test, the Point-of-Care Circulating Cathodic Antigen test (Rapid Medical Diagnostics, Pretoria, South Africa, Batch number: 170914118), a rapid test for intestinal schistosomiasis [29, 30](Fig. 6). Recording of the results was based on qualitative observation of color change of the test as per manufacturer instruction [29, 30]. Trace reactions of the test are considered positive [30]. After training, CHWs were provided with CCA test kits, urine collection containers, a copy of the job-aid (how to perform the test), examination gloves, disinfectants and disposal boxes. Community Health Workers (CHWs) were instructed to keep all the used POC-CCA tests (either with positive and negative results). Used test kits were collected on weekly basis and re-examined by a qualified laboratory technician as part of the quality check-up.

#### Praziquantel treatment coverage and drug inventory

In both arms, CHWs were provided with praziquantel drugs, praziquantel dose pole and treatment registry books. In the registry book, CHWs in the intervention arm, recorded the POC-CCA test results, the number of tablets/pills provided to each of the participant(s) based on the test results and any reported side effects. In the control arm, CHWs recorded the number of tablets/pills provided to each study participant and any side effects reported by participants.

**Fig. 7:**
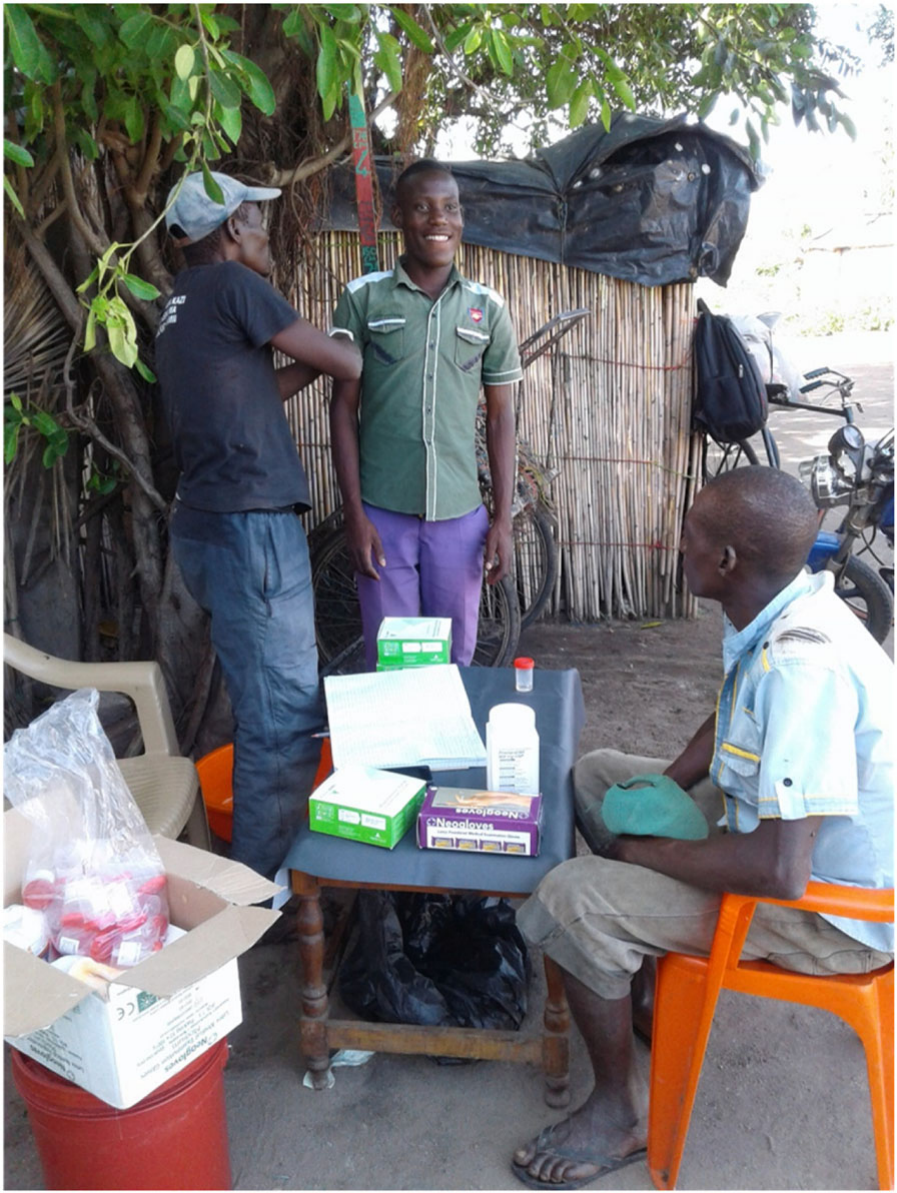
Community health workers in the intervention arms taking height of the study participant to decide on the number of praziquantel pills to prescribe to participant after diagnosis at Kome Island, north-western Tanzania

Diagnosis of intestinal schistosomiasis and provision of PZQ drugs was done at an agreed site within a cluster. Tablets were swallowed under direct observation (DOT) of CHWs. Estimation of number of PZQ tablets given to each participant was done using the praziquantel dose pole, based on the height of an individual (Fig. 7). Study participants who experienced any mild adverse side effects were counselled at the treatment premises and cases with severe adverse effects, were referred to the nearby health facilities.

#### Assessment of the community acceptability of the use of point-of-care circulating cathodic antigen by community health workers

A qualitative research approach using Focus Group Discussions (FGDs) was used to assess community acceptability of the approach. Eight community clusters out of 30 community clusters were selected (6 in each intervention arms and 2 control arms). In each of the cluster, one (1) FGDs session was conducted giving a total of 8 FGDs sessions. Each FGD consisted of 8–10 participants in each cluster. These FGDs participants included only those who participated in the study from the two arms. Focus Group Discussion participants were mobilized by CHWs from their respective cluster community and FGDs was conducted at a selected area near the cluster community. The interview guide was used as a tool to collect information on trust and confidence in CHWs, community willingness and acceptability of both approaches. For the intervention arm, additional information was collected on CHWs use of CCA rapid tests and trust of the test results from CHWs, compliance to treatment given and stigma associated with requesting urine samples from adult participants. The interviewer and the note taker were social scientists, speaking Kiswahili and the vernacular fluently. Table 4 shows the key questions which were used to stimulate the discussion.

**Table 4:** Key questions which were used to stimulate discussion with community members in the intervention and control arms

In the intervention arms	
1	How did you feel, being diagnosed by a trained person you knew?
2	How did you feel when the community health worker asked you to bring urine samples for diagnosis?
3	Did you feel any stigma when asked by the community health worker to submit urine samples for diagnosis of intestinal schistosomiasis?
4	How did you feel when the community health workers told you that you were infected with intestinal schistosomiasis?
5	Did you agree with the test results or did you have any doubt on the test results? If you had any doubt, what were your concerns on the results given?
6	After being told you had the infection and given drugs, did you take/swallow the drugs given?
7	Did you experience any mild side effects after taking the drugs administered by the community health worker? If yes, what were the side effects experienced?
8	In general, what are your perceptions on the test or the exercise of diagnosis and treatment offered by community health workers?
In the Control arm	
1	How did you feel to be treated/receive drugs without being diagnosed if you had intestinal schistosomiasis?
2	How did you feel when the community health worker used a praziquantel pole to decide on the dosage or number of drugs to give?
3	Did you agree to take the number of pills given after the community health worker measured your height and decided on the number of pills without diagnosing you if you had intestinal schistosomiasis?
4	Did you experience any mild side effects after taking the drugs administered by the community health worker? If yes, what were the side effects experienced?
5	In general, what are your perceptions on the mass treatment without diagnosis test or the exercise of treatment without diagnosis offered by community health workers?
6	What are your recommendations on the exercise of treatment without diagnosis offered by community health workers at your village/community?

#### Exploring the experience of community health workers after using point-of-care circulating cathodic antigen rapid tests

Focus Group Discussions with CHWs were conducted at the end of the study. A guide was used to stimulate the discussion and the discussion focused on their experience on using the CCA kits and provision of treatment after diagnosis, what were the challenges during MDA, what were the opportunities and what were the perspectives of the participants on the exercise, participants trust of the results and their recommendations to improve the MDA exercise with and without a diagnostic component. Table 5 shows the key questions which were used to stimulate the discussion.

**Table 5:** Key questions which were used to stimulate discussion with community health health workers in the intervention and control arms

In intervention arm	
1	What are the experiences and challenges you faced during the diagnosis and treatment of intestinal schistosomiasis exercise?
2	What opportunities did you take advantage of?
3	What are the perspectives of other community members regarding the test/diagnosis and treatment?
4	What recommendations do you have to help improve the test and treat exercise?
In control arm	
1	What are the experiences and challenges you faced during the treatment of intestinal schistosomiasis without diagnosis?
2	What opportunities did you take advantage of?
3	What are the perspectives of other community members regarding the treatment exercise without diagnosis?
4	What recommendations do you have to help improve the treat exercise without diagnosis?

#### Assessment of safety and accuracy of the circulating cathodic antigen (CCA) test performed by community health workers

A trained observer visited each of the CHW in the intervention arm at one, two and six weeks post-training and used a standardized checklist (Tables 4 and 5) to assess the performance of each CHW in adhering to safety procedures and procedures of performing the test, interpretation of the test results and recording of the test result. The observer also collected all the tests kits used within the week and sent them to a second observer to evaluate the test results. In addition, the field observer took a photograph of 20 POC-CCA tests with positive, negative and invalid result to assess CHWs ability to read CCA test accurately.

Observers were trained on how to use the check list and instructed on how to minimize observer bias. Using the checklist, the observer noted if CHWs performed each step correctly and also noted if she/he missed observing any step. The steps were divided into two parts, (i) those critical to safety and (ii) accurate performance of the test. Safety was defined as correctly wearing gloves, use of new urine container for every participant and disposal of the urine containers in approved buckets and urine samples in the toilet immediately after use. Whereas accuracy was defined as following all the necessary steps correctly to arrive at a correct diagnosis (i). Collecting urine samples in an enclosed pipette and pouring it in a test kit well (ii). Dispensing 2–3 drops of clearing buffer into the sample well (if provided by the manufacturer) (iii). Waiting for 15 min before reading test results (iv). Reading test results correctly (v). Recording the results. At each observation, the observer recorded number of POC-CCA rapid test performed since the previous visit and collected the used test kits, number of positives and number of treated participants. In addition, the observer recorded any challenges or concerns experienced by CHWs.

#### Monitoring of project activities performed by community health workers

Process monitoring approach using structured and unstructured observations methods was used to assess if activities were implemented as per study protocol. Unstructured observations mainly focused on behavior of treatment activities if participants were asked or instructed to take food/meal prior to drug intake or coming to diagnosis and treatment points after taking meals. In addition, the process monitoring focused on assessing if treatment procedures were followed, the praziquantel dose pole was used properly, recording any adverse events was done, and how CHWs managed any reported adverse effects. A trained observer and field coordinator were involved in process monitoring.

### Quality assurance plan

The CHWs in both arms were supervised after every one weeks by field-based supervisors and observers to detect and correct any deviations from the protocol. During the visit, the supervisor and observer assessed completeness of data captured in the booklets, adherence to inclusion criteria, filling consent forms, performance of POC-CCA tests, PZQ drug administration to participants, POC-CCA test storage condition and assessment of the tests. After every two weeks during the study time, a review meeting was conducted to address any challenges. All treatment registers were reviewed and CHWs with any challenge/problem received support and re-training. The accuracy of CHWs in performing the POC-CCA test was assessed at each visit.

The questionnaires which was used for the study, was developed and piloted in 70 participants from villages outside the study villages. The focus of the piloting activity was mainly to check for reliability of the questions. Field research assistants were trained on how to administer the questionnaire. For the qualitative data collection, an experienced social scientist was involved in training research assistants on the objectives of the study, how to take notes and how to conduct focus group discussions. Discussion guidelines were piloted to ensure that the meaning of the questions was clear to participants. Some questions were adapted from previous studies [31, 32]. All the FGD were recorded using digital recorders after obtaining consent from participants. The FGD were conducted in the Kiswahili language and after data collection, transcription and translation into English language was done. Selected transcriptions were checked to ensure quality of the transcription and translation and was shared with the team of social scientists for critical review.

### Data management and analysis

Some parts of the open checklist were coded before entered in the computer for analysis. Collected data were double entered using CSPro and the final data set were stored in MySQL database. Data were screened for consistency and errors corrected. Data analysis will be done using Stata version 15 (Stata Corp, College station, Texas, USA). Descriptive statistics will be used to describe participant’s demographic information. Assessment of uptake of praziquantel treatment will be done by comparing proportion of the outcomes between clusters in the two arms. For the intervention arm, treatment coverage will be assessed based on number of individuals tested and received treatment after diagnosis as registered in the booklets. For the control arm, coverage for praziquantel treatment will be assessed from the CHWs booklets/registers.

Odds ratios (OR) and 95% confidence interval (CIs) will be calculated using random effect logistic regression analysis adjusting for clustering. For assessing safety and accurate use of CCA test by CHWs, percentage of total steps, crucial steps and no-crucial steps performed correctly will be calculated for each observation cycle and a generalized estimating equation (GEE) logistic regression model for panel data will be fitted to identify factors associated with correct performance of at least 90% of the total steps. In addition, pooled mean and median percentage for reading of the 20 photographic results will be calculated for each observation cycle.

Descriptive statistics will be calculated of FGD participant’s demographic variables and socio-economic characteristics. The narratives during the FGD were recorded using a digital audio recorder, transcribed verbatim in the language of the study participants (Kiswahili) and translated into English. This was followed by verification for accuracy by a second social scientist fluent in Kiswahili and English. The transcripts generated were then imported into ATLAS ti, version 8 and qualitative data analysis was done according to procedures described previously [32]. The focus of the analysis was to reflect thematic description using the principle of interpretative phenomenological analysis (IPA) [33]. The goal of IPA was to explore insiders’ opinions and beliefs related to their experience of a particular phenomenon [33]. Using this strategy, themes were considered to be direct representations of the phenomenon under study [33]. Such approach gives an opportunity to explore social cognition and assumes that one’s interpretations of her experiences reflect the true nature of phenomenon [33]. In our case, the focus was in the experience of CHWs on using the CCA kits and provision of treatment after diagnosis, what were the challenges during MDA, what were the opportunities and what were the perspectives of the participants on the exercise, participants trust of the results and their recommendations to improve the MDA exercise with and without a diagnostic component. A guide was used to stimulate the discussion and the discussion focused on their experience on using the CCA kits and provision of treatment after diagnosis, what were the challenges during MDA, what were the opportunities and what were the perspectives of the participants on the exercise, participants trust of the results and their recommendations to improve the MDA exercise with and without a diagnostic component. Guided by this approach, coding for identified themes was done using a three phase coding system. In the first phase of coding, the primary social scientist performed an initial scan of the transcripts imported in the ATLAS t.i. software and establish initial themes. In the second phase, the social scientist focused in connecting themes and finding link in the data. The third phase, the primary social scientist re-read the data and assign excerpts which illustrated the final themes. Narrative text were applied around the themes, with quotes used to illustrate the text and communicate its meaning to the reader [33].

### Ethical clearance and consent to participate

The study was approved by the joint Ethical and Review Committee of Bugando Medical Centre and Catholic University of Health and Allied Sciences (CREC/200/2017). The study received further clearance from the region, district administrative authorities and village authorities. Kiswahili translated informed consent forms were used to obtain consent from study participants. Before recruiting study participants, CHW describes the objective of the study to participants, the benefits and any involve risk for them to participate in the study. For illiterate participants, a thumb print was used to sign the consent forms after a clear description of the study objectives was given to participants. Further permission was requested from study participants and CHW to include their pictures/images during publication of the study findings. All pictures present in this methodology section of the study have received permission from all the study participants and the CHW involved in the study. The trial is number PACTR201804003343404 registered at Pan African Clinical Trial Registry.

## Discussion

The overall aim of the present study is to document the additional role of the community health workers to use the point-of-care circulating cathodic antigen test to diagnose community members participating in mass drug administration for intestinal schistosomiasis. The study focused on the adult population who are currently not included in the on-going national MDA exercise, and who are known to have very low compliance to treatment [16]. The introduction of a diagnostic component in the community-based MDA exercise is expected to improve the uptake of praziquantel drugs among adult individuals. This will have a positive impact by reducing the risk of transmission of the disease.

Secondarily, the study will demonstrate the ability of the CHWs to use the POC-CCA safely and accurately. During training, majority of the CHWs demonstrated ability to interpret and differentiate POC-CCA test kits with positive and negative results. However, they had challenges in identifying invalid and unused POC-CCA test, which necessitated re-training. In addition, through the use of CHWs to diagnosed intestinal schistosomiasis using the POC-CCA test(s), the study will allow precise estimate of the prevalence of intestinal schistosomiasis among adult individuals in the study setting.

The inclusion of qualitative studies will help to acquire knowledge about the community perceptions and CHWs experiences after participating in the study. The focus for the community is to understand their acceptability of the diagnostic component performed by CHWs and recommendation to improve the exercise. Community acceptability is crucial for sustainability of any health intervention. Learning from CHW’s experiences, challenges and recommendation after implementing the study will help in improving the ways to integrate diagnostic component to community-based MDA run by CHWs in endemic areas.

## Conclusion

If introduction of a diagnostic component in the community-based MDA will have a positive impact on drug uptake and compliance, this will provide stakeholders with important evidence on how community-based MDA can be made innovative by integrating a diagnostic component performed by community health workers. This will have appositive impact on the risk of transmission of the disease.

## Abbreviations

CCA: Circulating Cathodic Antigen
CHW: Community Health Workers
DOT: Direct Observed Treatment
FGD: Focus Group Discussion
GEE: Generalized Estimating Equations
MDA: Mass Drug Administration
NTD: Neglected Tropical Diseases
PACTR: Pan African Clinical Trial Registry
POC-CCA: Point-of-Care Circulating Cathodic Antigen tests
PZQ: Praziquantel
SSA: Sub-Saharan Africa
WHO: World Health Organization
